# Crowded Trades, Market Clustering, and Price Instability

**DOI:** 10.3390/e23030336

**Published:** 2021-03-12

**Authors:** Marc van Kralingen, Diego Garlaschelli, Karolina Scholtus, Iman van Lelyveld

**Affiliations:** 1 Aegon N.V., Aegonplein 50, 2591 TV Den Haag, The Netherlands; marc.vankralingen@aegon.nl; 2 Lorentz Institute for Theoretical Physics, Leiden University, Niels Bohrweg 2, 2333 CA Leiden, The Netherlands; garlaschelli@lorentz.leidenuniv.nl; 3 IMT School of Advanced Studies, Piazza S. Francesco 19, 55100 Lucca, Italy; 4 Econometric Institute, Erasmus University Rotterdam, Burg. Oudlaan 50, 3062 PA Rotterdam, The Netherlands; karolina.scholtus@gmail.com; 5 Data Science Hub, De Nederlandsche Bank, Spaklerweg 4, 1096 BA Amsterdam, The Netherlands; 6 Department of Finance, VU Amsterdam, De Boelelaan 1105, 1081 HV Amsterdam, The Netherlands

**Keywords:** crowded trading, tail-risk, financial stability, entropy, G02, G14, G20

## Abstract

Crowded trades by similarly trading peers influence the dynamics of asset prices, possibly creating systemic risk. We propose a market clustering measure using granular trading data. For each stock, the clustering measure captures the degree of trading overlap among any two investors in that stock, based on a comparison with the expected crowding in a null model where trades are maximally random while still respecting the empirical heterogeneity of both stocks and investors. We investigate the effect of crowded trades on stock price stability and present evidence that market clustering has a causal effect on the properties of the tails of the stock return distribution, particularly the positive tail, even after controlling for commonly considered risk drivers. Reduced investor pool diversity could thus negatively affect stock price stability.

## 1. Introduction

This paper studies the effect of market clustering on price instability. We define market clustering as the degree to which groups of investors trade similarly. For each stock, our market clustering model measures the degree of trading overlap among any two investors that trade that particular stock. In general, stock prices are thought to adjust continuously to changes in the fundamental value of the stocks. The reactions of investors to new information determine the adjustments of prices and the resulting price dynamics. Market clustering, however, cannot be observed by individual investors and its effect on price dynamics can thus unfold unexpectedly.

Market clustering can be seen as a measure of the homogeneity of the investors’ pool. Reduced diversity of the investors’ pool, i.e., when the investors show similar trading behavior, means that coincidental overlap of trading strategies is more likely and overlap of trades increase the chance of crowded trades and overreactions, reflected in price fluctuations. The use of large-scale granular trading data and a novel complex network method enables us to study the effect of market clustering on price fluctuations directly. To the best of our knowledge, this is the first direct empirical investigation of the relation between market clustering and price fluctuations on individual stock level.

Studying the empirical relation between market clustering and price instability is relevant from both an academic and a supervisory point of view. First, the existing empirical literature on the topic focuses on only indirect measures of group behavior: overlapping portfolios [1,2], similarities in performance dynamics [3,4], dynamics of the number of owners per stock [5], or buyer and seller volume imbalance [6,7]. The suggestion that price fluctuations originate from uncoordinated or inefficient interaction among investors seems obvious, but due to limited data and lack of suitable methods, such effects have not yet been investigated directly.

Second, knowledge about the implications of market clustering is relevant for regulators, as market clustering can be an amplifying spillover channel for asset price fluctuations. The general implication of a causal relation between market clustering and price instability is that trading patterns through which investors react to incentives, matter for the efficiency of price discovery. Although this research focuses on the effect of market clustering on single stocks, market clustering might be a channel of volatility spillovers, because portfolio adjustments concerning other stocks in reaction to an initial price shock are more likely to overlap as well in a clustered trading environment. Therefore, market clustering might not only be a source of price instability, but also a channel of volatility spillovers, eventually resulting in correlated price jumps. In that case, market clustering would foster systemic risks. Market clustering might be an example of an existing market structure that can amplify seemingly unimportant events into widespread market volatility. In case market clusters coincide with otherwise interconnected institutions, for example, banks, common asset devaluation can be a crucial default contagion channel, as suggested in recent interdisciplinary research [8,9].

Market clustering is expected to cause price shocks, because it amplifies the effect of existing sources of price fluctuations. More specifically, market clustering is expected to increase the chance of price shocks in two different situations: Firstly, when the order deluge due to the group behavior overwhelms the supply [10,11] and, secondly, when the supply is thin due to the homogeneity of the investors’ pool, i.e. a lack of liquidity at one side of the order book [12]. In both situations market clustering increases the chance that the demand exceeds the supply, either in buy or sell orders.

We start our investigation of the influence of trading patterns by studying the relation between market clustering and the price dynamics of individual stocks. Our market clustering measure is unique in the sense that it quantifies two aspects of group behavior: clustering and crowdedness. We define price instability as an increase of the number of sharp price fluctuations, such that the tails of the log return distribution are heavier. Specifically, we investigate whether there is a causal relation between market clustering and the skewness, kurtosis, tail indices, positive and negative outlier counts, changes in downside risk, and upside gains.

The analysis of trading patterns depends on the ability to distinguish to what extent the observed patterns are the result of genuinely higher-order mechanisms, like group behavior, rather than of lower-order constraints. In this research, we represent a given trade configuration as a bipartite network, i.e., a two-layer network where stocks are represented as nodes in one layer, investors as nodes in the other layer, and links can only connect nodes across the two layers. These links represent the trades during a particular time period. We use the maximum-entropy principle to generate a null model with the same lower-order properties as the empirical network, in this case the so-called “degree sequence” (i.e., the vector containing the numbers of investors per stock and the numbers of stocks per investor). Certain “apparent” network patterns can actually be explained by the lower-order properties. Observations that deviate from the random network ensemble are instead indications of higher-order trading patterns. Our approach builds on recent research on the topological structure of economic and financial networks showing that the degree sequence explains the occurrence of several higher-order structures in these networks, while still being a local property that directly reflects the intrinsic heterogeneity of market participants [13,14,15].

Our source data consist of granular trade-by-trade records of Dutch banks and investment funds. These data are reported under the Markets in Financial Instruments Directive (MiFID). The data available to us contain all the transactions in stocks and bonds traded by all Dutch banks and investments firms (approximately 50). These trades are either conducted as an agent or for own account. The set of investors per stock is incomplete, as trades are only reported in this data set if a Dutch bank or investment firm is involved and hence we do not observe trades between two foreign parties.

The results indicate that the prices are less (more) stable for high (low) market clustering. We find evidence for a consistent and robust positive relation between market clustering and the kurtosis of the log return distribution. Clustering thus seems to be related to large price movements. Furthermore, we find a relation between market clustering and the tail index and outlier count for the positive tail, but interestingly not for the negative tail. We hypothesize that the effect for the negative tail is conditional on volatility state in the market and test this hypothesis with the dynamic panel data approach.

We use the data limitation of not observing trading among foreign investors to mimic an experimental research design and study the causality of the relation between market clustering and price instability. Per stock we measure what percentage of its turnover is traded by investors included in the MiFID data set and compare the results for stocks that are mainly traded by included investors (“treatment group”) with the results for stocks that are mainly traded by investors elsewhere (“control group”). Under assumption that Dutch and foreign investors trade in stocks with comparable properties, here we find evidence for causality as our results do not hold for stocks mostly traded by non-Dutch investors.

Finally, we examine market clustering and price instability in a dynamic panel data framework. Dynamic panel data models can account for heterogeneity bias across individual stocks and can disentangle causality effects in the presence of simultaneity driven endogeneity. These models show that clustering is a persistent process, affected by market conditions, but not by stock return momentum or fundamental variables. The only stock-related variables that matter are liquidity and market capitalization. Higher illiquidity in low volatility periods leads to higher clustering scores, indicating the investors are willing to take extra risks in low volatility periods. The model confirms the possibility that crowded trades are related to fire sales as less liquid stocks are traded more in downward markets. The relation between market capitalization and market clustering supports the presence of flight-to-safety within equities in turmoils. Thus the results are consistent with multiple equity market phenomena.

When we investigate the drivers of changes in Value-at-Risk (VaR) and Value-at-Luck (VaL; upside potential, measured as VaR but for the positive side of the return distribution), we find that our proposed clustering measure has explanatory power beyond other well-known variables. Our conditioning variables include practically all the variables suggested in the literature (i.e., market factor, book-to-market, dividend yield, size, Amihud (2002) liquidity measure, momentum, and market conditions). The findings confirm that stocks’ involvement into crowded trades lead to larger price fluctuations. The effect is stronger for the positive tail (VaL) and consistent with results from group comparisons. For the negative tail, market clustering causes price instability during financial turmoil, but not during calm periods.

The setup of the remainder of the paper is as follows. First, we provide a brief overview of the relevant literature. Then, we turn to a description of the data, followed by an explanation of the method to measure market clustering we developed. To the best of our knowledge, both the data and the method are new contributions to the literature. We then describe our results and close with a discussion.

## 2. Literature Review

The literature studying price dynamics is rich and can be classified in many ways. Our focus here is on joint trading affecting the market in such a way that it is no longer capable to perform two key functions: efficient price discovery and providing liquidity [16]. Several related strands of the literature shed light on this important issue covering (1) similar shocks on the funding side, (2) overlapping portfolios, (3) exogenous requirements, (4) market microstructure design issues, and (5) complexity models.

First, some argue that participants in the market face very similar funding shocks or, more generally, that investment needs or beliefs are highly correlated. This affects prices because leverage cycles result in fat tails [17]. For instance, Gorban et al. [18] suggest a continuous-time model where beliefs of strategic informed traders about crowdedness of trades and strategies in the market can lead to reduced liquidity on supply side and lower market depth.

Second, given the investment needs and outlook, investors will have accumulated a portfolio of assets that might to some degree be overlapping. With homogenous agents and perfect information, all portfolios will approach the market portfolio. In practice, investors are heterogeneous and information is uncertain and not freely available, thus investors will have portfolios that overlap only partly. This does not limit itself to liquid investments but also applies to longer term and less liquid exposures such as in the syndicated loan market [19].

Common asset holdings have attracted considerable attention, especially in the context of fire-sale spillovers and cascade dynamics [20,21,22]. Not surprisingly, studies find that more commonality in investments increase systemic risk with an exception to Barroso et al. [23], who discover no evidence of the relation between momentum crashes and institutional crowding. Gualdi et al. [24] show that portfolio overlapping on aggregate level increased slowly before the 2008 crisis, reached a peak at the start of the crisis and then triggered fire sales. Moreover, network effects are generally important (although Glasserman and Peyton Young [25] come to the opposite conclusion). Theoretical work has evolved from analyzing the effect of fire sales on a single portfolio and a single asset [26] to continuous time models with endogenous risk and spillover from fire sales across multiple assets and multiple portfolios [27]. Empirical (stress test) exercises assess how relevant such contagion effects are in practice. The results are highly dependent on the financial system considered (see, for example, van Lelyveld and Liedorp [28] and Cont and Schaaning [29]).

A third area of the literature relevant for our analysis highlights fire sales caused by an exogenous requirement. Note that fire sales are forced sales in stressed markets under unfavorable terms and are very different compared to regular buying and selling to adjust a portfolio. External requirements are often set by regulators to safeguard sufficient buffers for various risks (credit risk—using both risk weighted and risk insensitive measures (i.e., leverage ratios), counterparty credit risk, or liquidity risk [17,29,30,31,32].

Regulatory requirements often imply cliff effects as breaching certain thresholds come with costs. External demands leading to forced sales can sometimes also come from other market participants. For example, counterparties can call for margin. In particular, central clearing parties can require substantial margins to be delivered at very short notice [33].

Fourth, there is an established literature on mispricing because of market microstructure design and crowded trades [10,11,34]. Sometimes investors are prone to herding [35], at other times, speculators try to manipulate prices by rapidly submitting orders to drive up prices.

Finally, we develop and apply complexity models—as recently advocated by Battiston et al. [36]. Network theory in general has many applications in finance [8] and complex network theory offers reconstruction procedures and null models based on a maximization of entropy [13,14]. Such models have been applied to the world trade network [15] and banking networks [14,37,38,39]. A slightly different type of network emerges from order optimization as studied by Cohen-Cole et al. [40]. In studying the DOW and the S&P e-mini futures, they show that in these entirely electronic markets economically meaningful networks emerge. This happens despite the fact that the interjection of an order-matching computer makes social interaction impossible. In the method we develop here—to be elaborated on below—we incorporate the distribution of the number of links per node (degree distribution) but otherwise our expectation (or null model) is as random as possible.

**Figure 1:**
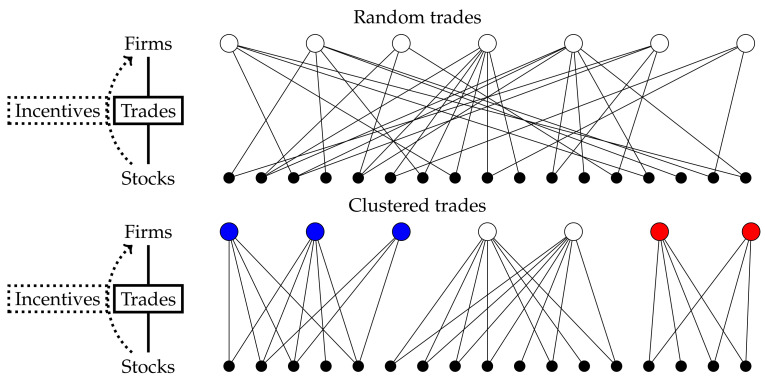
Market clustering in a bipartite network representation. The nodes in the top layer represent the firms and the bottom layer represents the stocks. The links between the layers represent all trades during a certain time period. Each line is a trade of the connected firm in the connected stock. In the top network, all trades are randomly distributed over the firms and stocks. The bottom trade network shows market clustering: Groups of firms trade in separated groups of stocks, while these stocks are traded only by these particular firms, which results in three distinct market clusters. The number of trades per firm and per security is the same for both random trades and the clustered trades example.

To clarify our approach to crowded trading, we present a graphical representation of market clustering in Figure 1. The homogeneity of the trading behavior of the investors’ pool per stock is then reflected in the market clustering measure that we will define below in Equation (1). In the most extreme case, the market breaks up into distinct submarkets, consisting of groups of investors that trade only in particular stocks which are only traded by those groups. Incorporating the effect of clustering into the measure on individual stock level is what sets our research apart from other crowdedness measures intended for individual stocks. For example, Yang and Zhou [6] differentiate between seller and buyer initiated crowded trades per stock. Their measure is based on trading volume data and thus does not reflect the (unobserved) interactions among investors. The same applies to the quarterly measure derived from mutual funds holding data in Zhong et al. [41]. The stocks that are largely held by actively managed mutual funds are classified as overcrowded but the tendency of a particular stock’s owners to trade with each other is not taken into account.

In general, peers trading similarly are likely to share common features, i.e., in case the group of investors that trade in a stock is very similar, then trading behavior might be similar, too. In our current analysis we abstract from what drives common trading. We are thus agnostic as to whether the order flows are driven by, for example, adjustments due to common asset holdings, (too) similar investment views, or shared regulatory constraints. Note that we do investigate what makes a particular stock attractive for involvement into clustered trades and how that depends on market conditions.

## 3. Methodology

In this section, we will first discuss our novel contribution: how to define a metric for homogeneous trading by comparing observed trade overlaps with expected overlaps under a suitable null model. We then introduce the definition of price instability and the cross-sectional comparison framework to assess the relation between clustering and price instability. Finally, we present a dynamic panel data model. We implement the latter in order to investigate the drivers of our newly defined measure as well as to show that it has additional explanatory power over and above well established covariates in models for downside risk and upside potential.

### 3.1. Measuring Homogeneous Trading

Our first goal here is to define a measure of similar or homogeneous trading behavior. This indicator will then be linked to the measures of price instability to investigate whether higher order patterns affect price formation. The nexus of trades between firms and stocks is complex and exhibits both lower- and higher-order network properties. Lower-order properties, such as the liquidity of a particular stock, have been researched extensively and are key determinants of price dynamics. Lower-order properties can be seen as the exogenous causes of price instability and their effects on price dynamics are direct and undelayed.

However, we focus on whether the market microstructure conceals particular grouping of trades that disturb the efficiency of the market. Particular ordering of the trades, resulting in higher-order patterns, can function as endogenous cause of price instability. Crucially, these market features are unobservable to the investors and their effects on prices can unfold unexpectedly. Such effects have not been investigated by use of granular trading data, because suitable methods had yet to developed and the data have been largely unavailable.

We develop a method that incorporates the information encoded, for each month *t*, in the number of unique investors per security (observed “degree” $d_{s,t}^{\mathrm{obs}}$ of security *s* in month *t*) and the number of unique, traded securities per firm (observed “degree” $d_{f,t}^{\mathrm{obs}}$ of firm *f* in month *t*). The observed degrees of all firms and securities during month *t* are combined into a vector $D_{t}^{\mathrm{obs}}$ representing the degree sequence observed in month *t*. We compare the observed trading network to a maximally random (i.e., maximum-entropy [13]) network ensemble based on only the observed degree sequence. The ensemble is characterized by a different connection probability $p_{sf,t}$ for each security-firm pair (s,f) and for each time *t* and, consequently, for combinations of links (i.e., “motifs”). Empirical deviations from the maximum-entropy ensemble are indications for higher-order patterns such as peers clustering in the same (type of) stock.

To identify market clustering, we need the observed values and the expected values based on the benchmark model. The quantity that represents the market clustering of security *s* during month *t* is

(1) $$m_{s,t}=\frac{M_{s,t}}{\langle M_{s,t}\rangle }-1,$$

where $M_{s,t}$ is the observed market clustering and $\langle M_{s,t}\rangle$ is the expected value based on the maximum-entropy model that we develop below. The observed value $M_{s,t}$ is divided by the expected value $\langle M_{s,t}\rangle$, so that deviations from the benchmark are scaled in terms of the expected value. The minimum value for the market clustering is minus one by definition and a market clustering of zero means that the market clustering has the same value as the expected value $\langle M_{s,t}\rangle$.

**Figure 2:**
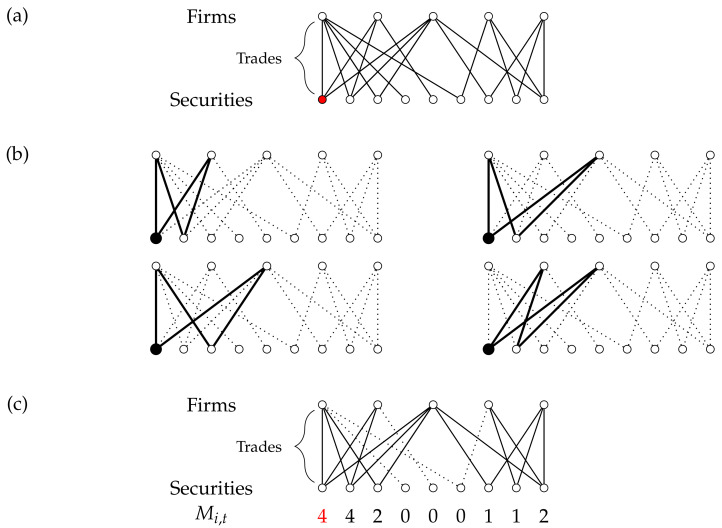
Example of the calculation of the observed market clustering $M_{s,t}$. (**a**) A hypothetical bipartite trading network. Each line represents a buy or sell transaction. (**b**) Counting the market clustering motifs for the first security. In these four cases shown the trading pattern exist and therefore the first security has score four. This calculation is repeated for all other securities. The summation in Equation (2) runs over all possibilities. (**c**) The same hypothetical trading situation with the observed market clustering $M_{s,t}$ for each security (lines that do not contribute to the market clustering measurements for any security are dotted).

The observed market clustering $M_{s,t}$—visualized in Figure 2—is defined, for each security *s* and month *t*, as the number of shared securities (other than *s*) traded by all pairs of investors trading in *s*. In other words, for each pair $\left(f,f^{\prime }\right)$ of firms, we first establish if they both trade in the security *s* during month *t*. If this is the case, we then count the number of securities (other than *s* itself) that these two firms are also trading simultaneously in the same month. The observed value of the market clustering $M_{s,t}$ for security *s* during month *t* is then given by

(2) $$M_{s,t}=\sum_{f}^{n_{F,t}-1}\sum_{f^{\prime }=f+1}^{n_{F,t}}(a_{sf,t}a_{sf^{\prime },t}\sum_{s^{\prime }\neq s}a_{s^{\prime }f,t}a_{s^{\prime }f^{\prime },t}),$$

where the total numbers of firms and securities active in month *t* are denoted by $n_{F,t}$ and $n_{S,t}$, respectively. The summation $\sum_{f}\sum_{f^{\prime }}$ runs over all possible pairs of investors and the summation $\sum_{s^{\prime }\neq s}$ runs, per pair of investors, over all securities except security *s*. The indicator $a_{sf,t}=1$ in case firm *f* trades in security *s* during month *t* and $a_{sf,t}=0$ otherwise. $M_{s,t}$ measures all trading combinations within the pool of investors that trade in security *s*, forming a market clustering pattern or “motif”. If investors in a security are otherwise not trading jointly, then $m_{s,t}=-1$ and we drop 3412 observations (5%) of such cases as these observations are not relevant for our analysis.

**Figure 3:**
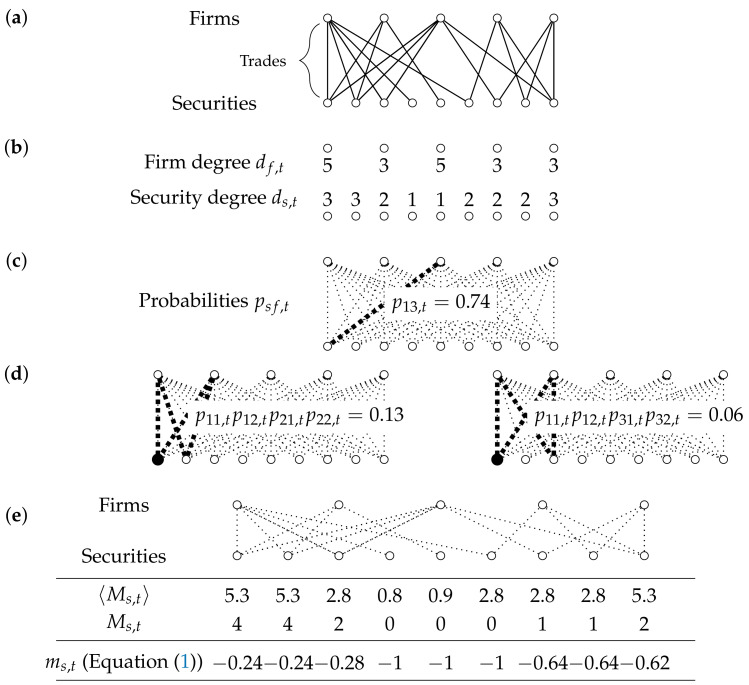
Calculation of the benchmark model for the market clustering $\langle M_{s,t}\rangle$. (**a**) The same hypothetical trading situation as in Figure 2. (**b**) The trading information is reduced to the degree sequence: The number of traded securities per firm and the number of trading firms per security. (**c**) The degree sequence is translated into a probability $p_{sf,t}$ for each firm–security pair (i.e., the probability of firm *f* trading in security *s* in month *t*). The probability and degree sequence hold the same information, as the expected value of the number of connections for each node equals the degree. (**d**) The probability of occurrence of the market clustering motifs equals the product of the four probabilities between the four involved nodes (see in Equation (3)). The expected value of the market clustering per security is the sum of all probabilities for motifs that are connected to the security. The first two motifs for the first security are shown. This calculation is repeated for each security. (**e**) The benchmark model market clustering $\langle M_{s,t}\rangle$ for each security, the observed market clustering from Figure 2, and the final market clustering measures, according to Equation (1), respectively.

We calculate the expected value of the market clustering based on the maximum-entropy probability distribution $P(X_{t}|D_{t}^{\mathrm{obs}})$ derived in Appendix A based only on the observed degree sequence $D_{t}^{\mathrm{obs}}$. As shown in Appendix A, the distribution $P(X_{t}|D_{t}^{\mathrm{obs}})$ factorizes over pairs of edges, which are all mutually independent in the null model. The expected value of the market clustering is therefore easily calculated as the sum over all configurations weighted by the probabilities:

(3) $$\begin{array}{ccc} \langle M_{s,t}\rangle \; & = & \sum_{X_{t}\in G_{t}}P\left(X_{t}|D_{t}^{\mathrm{obs}}\right)M_{s}\left(X_{t}\right)\\ \; & = & \sum_{X_{t}\in G_{t}}P\left(X_{t}|D_{t}^{\mathrm{obs}}\right)\sum_{f}^{n_{F,t}-1}\sum_{f^{\prime }=f+1}^{n_{F,t}}\left(a_{sf}\left(X_{t}\right)a_{sf^{\prime }}\left(X_{t}\right)\sum_{s^{\prime }\neq s}a_{s^{\prime }f}\left(X_{t}\right)a_{s^{\prime }f^{\prime }}\left(X_{t}\right)\right)\\ \; & = & \sum_{f}^{n_{F,t}-1}\sum_{f^{\prime }=f+1}^{n_{F,t}}\left(p_{sf,t}p_{sf^{\prime },t}\sum_{s^{\prime }\neq s}p_{s^{\prime }f,t}p_{s^{\prime }f^{\prime },t}\right), \end{array}$$

where we have introduced the single security-firm pair connection probability $p_{sf,t}$, defined as

(4) $$p_{sf,t}=\sum_{X_{t}\in G_{t}}P\left(X_{t}|D_{t}^{\mathrm{obs}}\right)a_{sf}\left(X_{t}\right)$$

(see in Appendix A for a detailed calculation of $p_{sf,t}$ from $D_{t}^{\mathrm{obs}}$) and exploited the fact that, under the conditions $s\neq s^{\prime }$, $f\neq f^{\prime }$ guaranteed in Equation (3),

(5) $$\sum_{X_{t}\in G_{t}}P\left(X_{t}|D_{t}^{\mathrm{obs}}\right)a_{sf}\left(X_{t}\right)a_{sf^{\prime }}\left(X_{t}\right)a_{s^{\prime }f}\left(X_{t}\right)a_{s^{\prime }f^{\prime }}\left(X_{t}\right)=p_{sf,t}p_{sf^{\prime },t}p_{s^{\prime }f,t}p_{s^{\prime }f^{\prime },t}$$

due to the independence of distinct edges. Figure 3 illustrates the summation process graphically.

**Figure 4:**
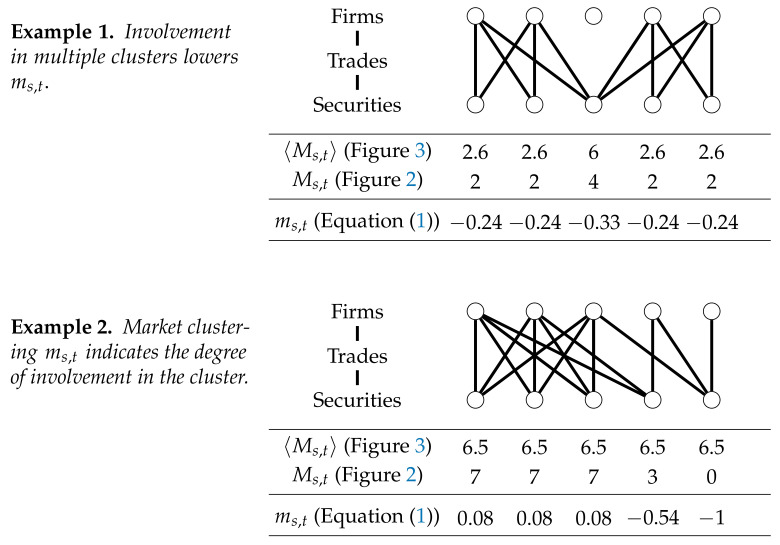
Example of the computation of $m_{s,t}$. Example 1. The market clustering is lower when a security is involved in multiple clusters at once. In this configuration two clusters exist: one on the left and one on the right. The security in the middle is involved in both clusters. The final market clustering is lower for the security in the middle, because its four connected firms are not mutually clustered. Example 2. The market clustering $m_{s,t}$ indicates the involvement in the market cluster. All securities are traded by three firms each. The left three firms are almost fully clustered. The final market clustering $m_{s,t}$ indicates to which extent the securities are involved in the cluster.

The market clustering $m_{s,t}$ measures the degree of clustering for security *s* among its traders. Figure 4 shows examples of the performance of the method in two hypothetical situations. First, the model assigns a lower value to securities which are involved in multiple clusters. Arguably, the involvement in multiple clusters enhances the diversity of the investors group and would probably stabilize the price dynamics. Second, the work in Figure 4 shows that the model is able to indicate to what extent the security is involved in the cluster. Homogeneous trading behavior is indicated by a relatively high percentage of overlapping trades. Therefore, the number of trades that do not overlap must lower the market clustering measure. This condition is satisfied as can be seen in the second example in Figure 4.

### 3.2. Measuring Price Instability

We measure stock price instability with statistics that focus on tail behavior of the stock return distribution. We analyze the skewness, the kurtosis, the tail indices, the number of outliers, and the changes in the left and right 5% quantiles. The latter two can also be interpreted as changes in downside risk and upward potential and are better manageable on the time-series dimension. Ang et al. [42] show that sensitivities to downside market movements are priced in addition to the common risk factors. Thus, if market clustering leads to changes in downside risk, it implicitly shows up in the price dynamics.

Skewness and kurtosis are measures of the shape of the complete log return distribution while the outlier count and the tail index are focused on the tails of the distributions—the extreme returns. The tail index (i.e., Hill’s estimator) measures the fatness of the tail according to the power law distribution. We count the number of outliers by sequentially applying the generalized Grubbs’ test until no outliers are detected. The skewness, Hill indices and outlier count also allow us to distinguish the effect on price instability for up- and downward shocks separately. We measure the size of the price fluctuation relative to the yearly standard deviation of the stocks, i.e., we divide the log returns by the yearly standard deviation per stock. Complementary to the volatility normalization, we investigate the influence of market clustering on the variance and the Median Average Deviation (MAD), which is more robust to outliers than the variance.

Value-at-Risk (VaR)—often used in risk management and regulation—is an obvious choice for quantifying the downside risk. We focus on a single stock 5% VaR obtained via historical bootstrap from daily returns. Historical simulation risk measures depend on the level of volatility in the sample. However, our quantile-based variable measures change over time and, as such, it is not affected by volatility clustering. More precisely, for the monthly data set we define

$$\Delta {\mathrm{VaR}}_{st}=100\left(\frac{{\mathrm{VaR}}_{s}\left(t-11,t\right)}{{\mathrm{VaR}}_{s}\left(t-12,t-1\right)}-1\right),$$

where ${\mathrm{VaR}}_{s}\left(t_{1},t_{2}\right)$ denotes a 5% VaR for stock *s* at the end of month $t_{2}$ obtained via historical bootstrap from daily prices over the period from month $t_{1}$ to month $t_{2}$.

Similarly, to capture tail asymmetries, we define changes in Value-at-Luck (VaL):

$$\Delta {\mathrm{VaL}}_{st}=100\left(\frac{{\mathrm{VaL}}_{s}\left(t-11,t\right)}{{\mathrm{VaL}}_{s}\left(t-12,t-1\right)}-1\right),$$

where ${\mathrm{VaL}}_{s}\left(t_{1},t_{2}\right)$ denotes a 95% VaR for stock *s* at the end of month $t_{2}$.

### 3.3. Stochastic Dominance and Causality for Groups

We now compare the distributions of the price instability measures for low and high market clustering. First, the securities are ordered according to their market clustering measure. Second, the securities are divided into three groups: the lowest (*L*) and the highest (*H*) 33%. We ignore the middle group in the remainder. Finally, we collect all time series price instability measures per time window per group and assess first and second order stochastic dominance of the distributions for group *L* and *H*.

We use three tests to indicate the differences between the distributions of groups *L* and *H*. The Kolmogorov–Smirnov (KS) test and the Mann–Whitney–Wilcoxon (MWW) test are both nonparametric tests for unpaired samples. The $\chi^{2}$ test is used instead of the KS test in case of binned data, because the KS test is unreliable when the number of ties is high. The KS test is sensitive to any discrepancy in the cumulative distribution function and serves as a test for the first-order stochastic dominance. The MWW is mainly sensitive to changes in the median and aids to evaluate the second-order stochastic dominance. We use visual inspection of the cumulative distributions to study the nature of the discrepancies to interpret the test results.

Using the difference-in-differences approach allows us to benefit from the partial coverage of our data set and dispel concerns over reversed causation. A concern could be that rather than market clustering causing price instability (null hypothesis), unstable and risky stocks might attract traders that prefer to trade in clusters of like-minded traders. In order to assess the effect of clustered trading in a mimicked experimental research setting, we construct a so-called control group from the stocks that are mainly traded by investors *not* included in the our data set. We look at the relation between market clustering and kurtosis in the control group. A significant relation would be speak against causality. The test is valid under an assumption that both groups of investors trade in stocks with somewhat similar properties.

### 3.4. Dynamic Panel Data Framework

The last part of the analysis applies a dynamic unbalanced panel data model. We aim to strengthen the high and low market clustering group results by exploring (a) the possible drivers of the clustering measure and (b) the effect that the clustering measure has on price instability. We tackle two questions in the model for the market clustering drivers. First, a group of investors may choose particular stocks because of their (latent) properties. We also include the properties that quantify a stock’s riskiness and instability as an additional test on reverse causality, mentioned in the previous section. Second, crowded trading activity may depend on certain market conditions. We look at the effect of the perceived trend and volatility. In the second application, we investigate the relation between changes in left and right quantiles of log returns distribution and clustering in individual stocks. In particular, we are interested to see whether a higher clustering measure leads to larger changes in downside risk VaR and upside potential VaL after controlling for other possible individual stock risk determinants.

The general representation of the model with both lagged dependent and independent variables included, possibly of different depth, is

(6) $$y_{st}=\sum_{r}\rho_{r}y_{s,t-r}+\sum_{p}\beta_{p}^{T}x_{s,t-p}+\alpha_{s}+\epsilon_{st},r=1,2,\cdots ,p=0,1,2,\cdots ,$$

where $y_{st}$ is a dependent variable, i.e., the clustering measure or the price instability measure depending on exact specification, $x_{s,t}$ is a vector of considered covariates, $\alpha_{s}$ is an individual effect, $\rho_{r}$ and $\beta_{p}$ denote model parameters, $\epsilon_{st}$ is idiosyncratic error term, s=1,⋯,N, and t=1,⋯,T.

We opt for the fixed effects model and treat $\alpha_{s}$ as a set of *N* additional parameters. We do not employ time dummies for two reasons: First, time dummies would preclude including time-only varying variables of interest (like the market factor MKTF and market volatility VIX). Second, incorporation of time dummies is more suitable for panels with very small *T*. In fact, most of our efforts to run the dynamic model with both fixed and time effects result in singularity issues. We estimate Equation (6) with the System GMM. In particular, a two-step estimator with Windmeijer [43] correction for standard errors is used. Estimation is carried out with the R package plm [44].

Our methodology has several attractive properties. First, individual effects are allowed to be correlated with the covariates $x_{st}$—a likely case in our data as, e.g., firms in certain industries may have higher dividend yields or price-to-book ratios than others. Second, the fixed effects approach accounts for unobserved heterogeneity bias. All (practically) static cross-sectional stock features, like sector or exchange, are by default incorporated into $\alpha_{s}$ terms. Last but not least, we address potential endogeneity issue due to simultaneity. We hypothesize that an increase in the clustering measure leads to larger changes in downside risk. However, it is also possible that some stocks are more likely to end up in cluster trades because of their risk profile. We disentangle the causality by producing internal instruments for the right hand side variable CLUST that is not strictly exogenous.

A common approach is to use all possible lags and variables to construct GMM-style instruments. Roodman [45] warns that too many instruments result in model validity issues and, specifically, false estimation outcomes and low power of overidentification tests. Roodman suggests collapsing the instruments and using only certain lags to overcome the instrument proliferation. Wintoki et al. [46] show that both collapsing the instruments and the size of cross section increase the power of Sargan-Hansen J test. We use all available lags for selected variables and construct collapsed GMM-style instruments.

## 4. Data

The data have been collected as part of the Markets in Financial Instruments Directive (MiFID). MiFID is a European Union (EU) law to regulate investment services across the European Economic Area (EEA). The directive applies to all firms that perform investment services and activities. Firms that only perform ancillary services are exempted. “Post-trade transparency” is the key aspect of MiFID mandating the authorities to collect the data used here. The post-trade transparency regulation requires all firms to report all trades in all listed stocks, including the time, the price, and number of units to the supervisory authorities immediately after the trade. MiFID only contains information about the transactions and thus holdings that are not traded are not in the data.

Although MiFID collects data on a EU level, Dutch authorities only have access to the transactions of Dutch banks and investment firms. In particular, the data cover the investments in financial instruments of 86 Dutch banks and investment firms. The time span of the data covers January 2009 through April 2015. The annual cross-sectional analysis (see Section 5) is thus done for the period January 2009–December 2014. Only the face-to-market firms report their transactions. The data contain trades by the reporter as principal trader and as agents. For the market as a whole, agent trades form a limited part and are roughly at 10% of volume/trades. Furthermore, although we do not have information on the identity of the clients, it is likely that they are non-financial firms or retail clients and hence will be very heterogeneous in their trading strategies. For the moment, we thus concentrate on trades entered into as principal. In case a principal trader performs transactions via a broker, only the broker reports the transaction, but we do see it in the data.

Contrary to portfolio holdings data sets, such as the ESCB Securities Holding Statistics, which show only shifts in the portfolio holdings; the MiFID data set contains all buy and sell transactions separately. We aggregate these transaction level data to a monthly frequency, split by the total number of buy and sell transactions. Aggregation of data is necessary because trading clusters do not emerge instantaneously, but rather over time. This choice facilitates our research design, meaning that we can derive price instability metrics from less noisy daily data instead of intra-day observations.

To improve the comparability of the price dynamics, we perform the cross-sectional comparison only for equities and exclude bond trading. In general, the price dynamics and trading behavior differ markedly between equity and bond markets. In contrast to equities, most bonds are not unique as bonds issued by the same entity, but of different maturities are to a degree interchangeable (in case no arbitrage opportunities exist). In addition, we want to abstract from the dynamics at the beginning or end of the lifetime of a security (e.g., an IPO or a default). Thus we select 976 equities that are traded during each month in the period January 2009–April 2015.

The data source for the daily stock return time series is Bloomberg Professional. In case securities in our data are traded at multiple exchanges, Bloomberg chooses between the exchanges automatically. In case no transactions are registered during the day, the price of the security is kept at the price of the last transaction. After inspecting the price series for outliers, we remove two time series of penny stocks with excessive return volatility.

We apply a panel data framework for securities classified as common stocks in the Bloomberg database. The initial sample of 976 equities contains 583 common stocks. We remove 16 stocks for which the average price does not exceed 1 EUR, then 24 stocks which are thinly traded (more than 10% of days during the trading period without a single transaction), 2 stocks with non-euro currency data, and 2 stocks with suspiciously large values for some fundamentals. Next, we apply the turnover requirements for each year as in the first part of the analysis. Many of the stocks qualify for multiple years, in total we have N=269 unique stocks and T=76 months. The number of stocks across years fluctuates between 203 and 234.

The summary of explanatory variables and applied transformations is shown in Table 1. We consider a wide variety of potential risk and trading behavior drivers: stock market conditions, individual stock performance, liquidity, and fundamentals. MKTF and VIX are only time-varying variables, LEV3 monthly values repeat for the same fiscal quarter, and all other variables vary per stock per time period. Non-time-varying variables, like the sector of the issuer, cannot be explicitly accommodated in a panel framework with fixed effects.

The Fama and French market factor for Europe is downloaded from the Kenneth French library (See https://goo.gl/pZVmqe (accessed on 18 January 2017)). The VIX index comes from Chicago Board Options Exchange website (See https://goo.gl/zMCTa (accessed on 15 March 2017)). We obtain all stock specific information via Bloomberg terminal.

Table 2 presents the descriptive statistics for variables in the panel data models. We discuss the last two columns as they offer the most valuable insights with regard to methodological choices. ΔVaR, the percentage point change in the VaR, has substantial within variation of 0.356, and thus the fixed effects model seems suitable for it. ΔVaL, the percentage point change in the upside VaR, has somewhat smaller yet acceptable within variation. We can expect effects of MKTF, VIX, and MOM to be estimated precisely because of (relatively) high within variation proportions of 1.000, 1.000, and 0.338. Perhaps we will see effects of CLUST and DY as well, but the rest of the variables are likely to have high standard errors. MCAP and ILLIQ have such high between groups variation (close to one) that their explanatory power may be subsumed by fixed effects.

Table 3 provides information about co-movements of the variables included. The clustering measure CLUST has significant though small correlations with most variables, except for MKTF. The largest correlation of 0.102 is observed with VIX, indicating that the level of clustering could be dependent on market conditions. CLUST has marginally significant positive correlations with changes in downside risk and upside potential, i.e., ΔVaR and ΔVaL, of 0.018 and 0.020, respectively. ΔVaR and ΔVaL are also strongly correlated with VIX. In the dynamic panel data models, we aim to disentangle the causality direction and the effect of general market volatility on both clustering and risk measures. Furthermore, there is zero correlation between the changes in 5% quantiles in the left and right tail of the distribution. As within variation for these variables is non-negligible, likely, they possess very different dynamics over time supporting our choice to estimate separate models for changes in the left and the right tail.

The autocorrelations and partial autocorrelations (Table 4) indicate the dynamic nature of all risk series and the clustering measure. Two lags seem an appropriate starting point for the dynamic models explaining CLUST, ΔVaR, and ΔVaL.

## 5. Results

### 5.1. Group Comparison

**Figure A1:**
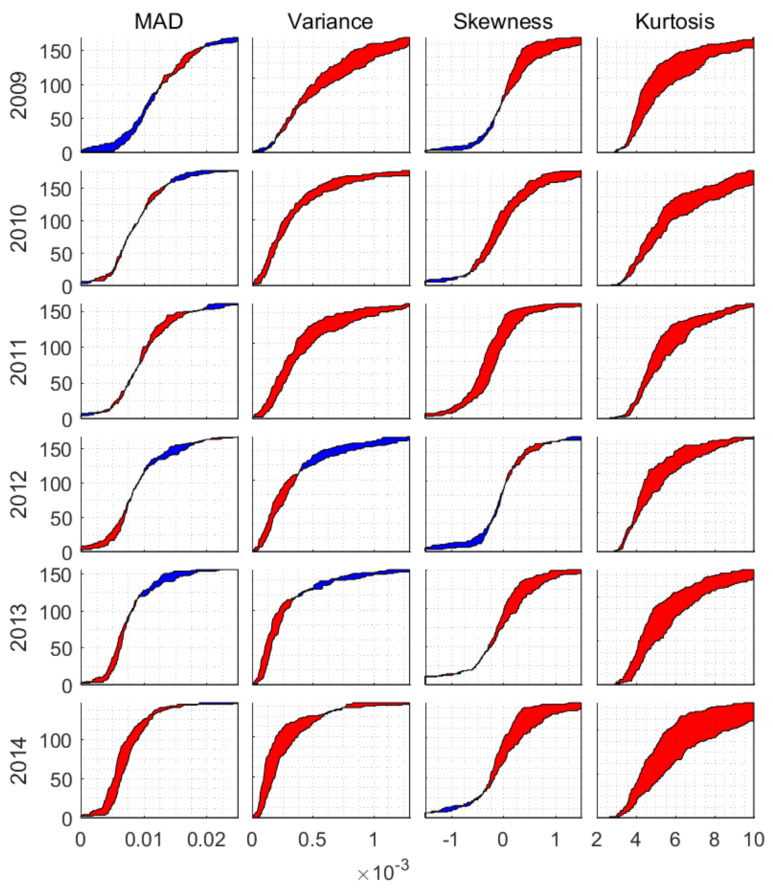
Cumulative distributions for low and high market clustering per time series measure (MAD, variance, skewness, and kurtosis) and per year. The space in between the distributions for low and high market clustering is colored to indicate which distribution is higher. Red means that distribution *H* (high market clustering) exceeds distribution *L* (low market clustering), and vice versa for blue.

We compare distributions of price instability measures between the buckets of stocks with high and low market clustering. Our first key observation is that there seems to be a relation between the kurtosis of the log return time series and market clustering. Table 5a shows an overview of the results of the 24 test cases (MAD, variance, skewness, kurtosis where in each cell we show the results of the Kolmogorov–Smirnov (KS) test, and the Mann-Whitney-Wilcoxon (MWW) test). For all six years, both tests give significant indication for a positive relation between market clustering and the kurtosis (with significance level of 2.5%). The test results are confirmed visually by the distance between the graphs of the cumulative kurtosis distribution for low and high market clustering (see Figure A1) (Appendix B). The cumulative distribution of the kurtosis in high market clustering group stochastically dominates the cumulative distribution of the kurtosis in low market clustering group. As the sample kurtosis is a measure of tail extremity and peakedness, the stocks with a higher (lower) market clustering tend to have log return distributions which are more (less) peaked and have (less) fat tails.

**Figure A2:**
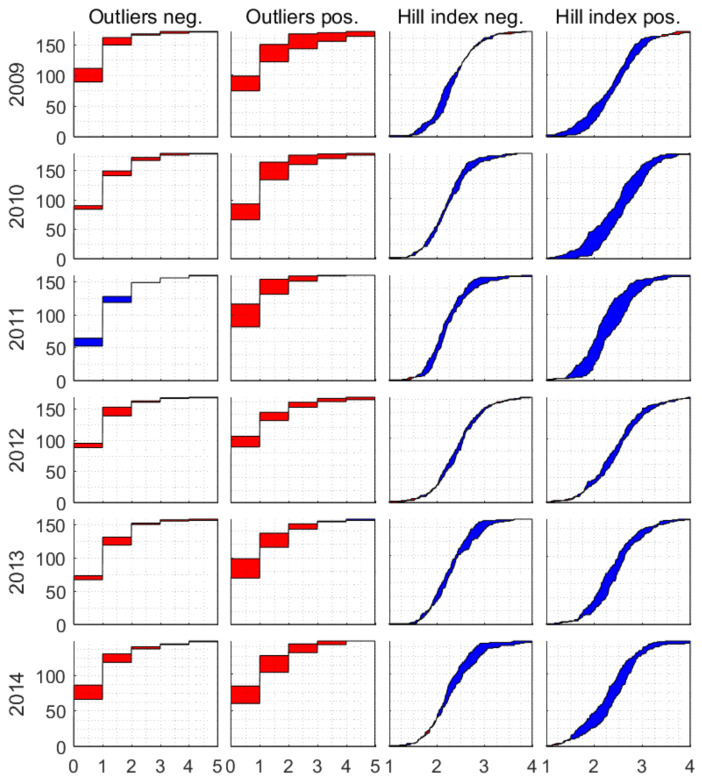
Cumulative distributions for low and high market clustering per time series measure (number of negative outliers, number of positive outliers, Hill index for the negative tail, and Hill index for the positive tail) and per year. See Figure A1 for explanation.

The stochastic dominance of distributions of considered price instability measures conditional on positive and negative tail in high vs. low market clustering groups indicate that market clustering relates to a relatively heavier tail for the positive tail of the log return distribution and not for the negative tail. The results for the Hill indices (Table 5b and Figure A2) show that only the fatness of the positive tail relates to market clustering. Distribution of positive tail index in low clustering group dominates distribution of positive tail index in high clustering group. Here, a lower index implies fatter tails. The results for the outlier count (Table 5c and Figure A2) also show a clear relation between the number of positive outliers and market clustering and not for the number of negative outliers. Distribution of positive outliers in high clustering group stochastically dominates distribution of positive outlier in low clustering group. The stochastic dominance of distributions of price instability measures for the negative tail cannot be established. The tests in Table 5 provide evidence for neither first nor second order stochastic dominance.

The positive relation between the skewness and market clustering in Table 5 and Figure A1 is in accordance with the observation that the market clustering relates to a relative increase of only the upward price fluctuations. However, this does not mean that the kurtosis results in Table 5 are solely caused by the upper tail. The robustness checks in Table A1 (Appendix C) for partial data show that the relation between market clustering and the kurtosis is also significant when the tail observations of the log return distributions are left out of the analyses. Furthermore, the lack of clear unconditional relation of price instability and market clustering in the negative tail does not preclude a possibility of a conditional relation. We investigate market conditions as a possible confounding factor in the panel data framework.

The significance of the relation between market clustering and price instability varies over time, as the test results for shorter time spans indicate. Table 6 repeats the results of Table 5a for a time window of two months. Approximately half of the kurtosis test results for a time window of two months are the same as in the yearly results. For 2009, Table 6 shows a clear positive relation between the kurtosis and market clustering. During the period 2010–2011, the positive relation seems to apply to the end of 2010 and the first half of 2011. In 2012 and the first half of 2013, no consistent relation exists for any of the measures or time window. For the end of 2013 until the end of the sample, the kurtosis results are mostly positive. The significance of the results at shorter time scales is reduced because the time series measures have a higher spread at shorter time scales, while the number of observations stays the same. The significance of the relation between market clustering and price instability might vary because the samples within the time windows are too small. Nevertheless, the relation between market clustering and the kurtosis is positive in more than half the test statistics for the two month time windows.

The results for the skewness, kurtosis, and outlier count are normalized by the volatility. We show the relation between the variance and market clustering separately in Table 5 and Table 6 and Figure A1. In addition, we analyze the results for the MAD. We find no consistent relation between market clustering and the yearly MAD. We find a weak but consistent positive relation between market clustering and the yearly variance. Figure A1 shows that the discrepancy between the distributions is smaller for the MAD and variance than for the kurtosis. The results for time spans of two months (see Table 6) show an increase in the MAD and variance during the periods where the kurtosis results are consistently positive. The relation between market clustering and the MAD and variance is not informative in itself, as the stocks are traded in different markets. The observation that more (less) market clustering relates to stronger (weaker) price fluctuations is in accordance with the observation that market clustering relates relatively more to the variance than the MAD, because the MAD is more robust to outliers than the variance. Market clustering relates also to price instability measured relative to time-varying volatility. Table A3 shows the relation between market clustering and the yearly kurtosis of log returns normalized by the conditional standard deviation estimated by various GARCH models. This indicates that the relation between market clustering and price instability is not confined to periods of high volatility.

Using the partial coverage of our data set we can dispel concerns over reversed causation. Rather than market clustering causing price instability, unstable stocks might attract traders that prefer to trade in clusters. If the latter holds, then the relation between kurtosis and market clustering would be independent of what percentage of the total turnover traded is included in the data set. Table 7 shows that the relation between market clustering and the kurtosis vanishes for stocks that are mainly traded by investors which are not included in the MiFID data set. The relation between the kurtosis and price instability is (not) significant for stocks with a high (low) percentage of the turnover traded within the data set. By difference-in-differences logic, these results indicate that market clustering leads to price instability and not the other way round.

### 5.2. Drivers of Market Clustering

An important question is whether our proposed clustering measure actually captures new, previously ignored information. To investigate which observable drive investors’ pool diversity we use a dynamic panel data framework. Estimated models, shown in Table 8, suggest that clustering is quite a persistent process. Thus, if at time *t* clustering is high (low), it is likely to be high (low) at t+1, too, mainly driven by commonalities, illiquidity, and size. Other stock specific variables have little to no effect in our setting. No more than 20% of the clustering measure variation can be explained by characteristics that would proxy for investor preferences. Thus, a large part of the clustering measure variation remains unexplained and is likely due to accidental portfolio overlap.

Table 8 demonstrates that crowded trading is a persistent feature as the clustering measure exhibits significant positive dependence on the lagged values of market clustering in all models. Herding, lasting for at least multiple months in upward markets, could be one of the mechanisms related to clustering. If market clustering results from accidental portfolio overlaps, continuing clustering may be observed due to spreading the orders over time to reduce market impact. The persistence of market clustering suggests the need for further research with adjusted measures of market clustering that differentiate between buy and sell orders. Furthermore, investigation of the stability of the investors’ pools involved in clustered trades would be helpful in understanding the effects of market clustering.

There is little evidence that individual downside risk affects the clustering measure. Lagged ΔVaR is marginally significant in Models 1 and 3; thus, there is not sufficient evidence to conclude that market clustering is stronger for the stocks with increasing downside risk. All of the three models in Table 8 consider downside risk as endogeneous variable in line with our hypothesis that market clustering causes price instability.

Market direction and market risk affect market clustering in multiple ways. First, all models indicate that increase (decrease) in market returns or market volatility in the previous month lead to significantly more (less) clustering per average stock. We added VIX to the GMM-style instruments to correct for potential VIX endogeneity, i.e., that clustering feeds aggregate market volatility. Second, lagged general market uncertainty (VIX${}_{t-2}$) has a negative effect. We interpret this as a short-term corrective mechanism: when increased market volatility leads to more crowded trades, then a month afterwards the trading subsides (because the funds are used up, the interest is transferred elsewhere, investors get scared of continuing uncertainty, or some other reason) and so do the clustered activities. Third, market conditions play a role through asymmetric effects of stock size and illiquidity on clustering measure. Model 2 looks at the effect for high and low volatility states, and Model 3 shows the differences across up and down markets. The specifics of these asymmetries and implications are discussed further in the next paragraph.

Illiquidity and size are the only two stock-specific variables that affect clustering, while momentum, price-to-book ratio, dividend yield, and leverage do not yield a significant coefficient in any of the models. To better understand illiquidity and size effects, we investigate asymmetries across market conditions (Models 2 and 3). We find that in quiet times market participants tend to cluster around less liquid stocks (significant coefficients for ILLIQlow and insignificant for ILLIQhigh), perhaps because they are willing to take more risks. Less liquid stocks end up in clustered trades in downward markets, too (Model 3). This resulting pattern is consistent with fire sales. When the stock owner’s pool is homogeneous and the pressure to sell arises due to, for example, margin calls, selling less liquid stocks leads to higher price impact, further fueling margin calls and stock sales. Market capitalization (MCAP) has (marginally) significant coefficients in Models 1 and 2. In high volatility markets large firms attract more crowded attention than they do in low volatility markets (coefficients of 0.734 vs. 0.367). Large stocks are frequently dividend paying, are likely index constituents, and are considered less risky, thus such trading behavior may be viewed as a flight-to-safety within equities.

To summarize the insights from this section, the results support theories that market clustering could be a consequence of multiple mechanisms. For one, herding induces persistence in the clustering measure time series. Next, willingness to take up more risks in low volatility markets and fire sales in downward markets both manifest as increased clustering around less liquid stocks. Finally, more clustered trades with higher market capitalization in high volatility period can be interpreted as flight-to-safety phenomenon. Interestingly, we see no evidence that stock selection based on fundamental characteristics would lead to market clustering.

### 5.3. Downside Risk, Upside Potential, and Clustering

We now turn to the causal analysis of market clustering and price instability. We employ a dynamic panel data model to analyze whether our newly proposed measure actually has additional explanatory power in modeling changes in the downside risk and the upside potential in addition to all commonly used conditioning variables (as discussed in Section 4). In short, we find that market clustering indeed causes price instability, but the effect is conditional on the volatility state in the market.

Table 9 contains the results. All of the models consider the price instability measure as an endogenous variable in line with our concerns that price instability could lead to market clustering. Models 1 and 2 look at changes in the downside risk, and Models 3 and 4 look at changes in the upside potential. All models include current and lagged (conditional) values of CLUST.

Consistent with the outcome of stochastic dominance analysis, there is no causal relation between CLUST and price instability in the negative tail (Model 1). Model 2, however, reveals that in high volatility markets the relation is significant. This makes crowded trading a dangerous phenomena, likely fostering contagion. On the positive side of the return distribution (Models 3 and 4), clustering leads to price instability in both high and low volatility periods. Based on the squared correlation between the dependent variable and fitted values, the positive tail is harder to explain, nonetheless. Lagged CLUST yields insignificant coefficients in all models; thus, the direct causation for the positive tail as well as for the negative tail is contemporaneous and we find no evidence of predictive relation.

Other coefficients have the expected signs or are insignificant. Strongly significant variables come from two categories: aggregate market related (MKTF and VIX) and derived from returns (MOM, MCAP, and ILLIQ). Upward movement and trend in the market index lead to smaller individual risks (thus negative changes in VaR) and more gradual price increases (thus slightly negative changes in VaL). Current increase in volatility also increases changes in downside risk. For the positive tail of the distribution we again see the short-term corrective mechanism: higher volatility at time *t* implies higher average change in upside potential, but taking advantage of this will result in reducing the upside potential for the next period. Positive momentum, higher market capitalization, and higher illiquidity have negative effect on the changes in log return distribution quantiles. Fundamental characteristics (PB3, DY, and LEV3) do not consistently contribute to explaining the time variation in positive and negative quantiles of return distribution.

All in all, we show that market clustering causes contemporaneous price instability. The relation is present in the negative tail during turmoil and in the positive tail independent of the volatility level.

## 6. Discussion

We have shown some suggestive evidence for a causal relation between market clustering and price instability on the individual stock level. There seems to be a consistent and robust positive relation between market clustering and the kurtosis, the skewness, the positive tail index, the positive outlier count, and the right 5% quantile of the log return distribution. The positive relation between market clustering and the left 5% quantile of the log return distribution is conditional on periods of high volatility. Focusing on extreme price fluctuations, that is, the tails of the normalized log return distribution, we find that market clustering generally causes an increase of large upward price shocks. Increases of large downward shocks due to market clustering turns out to be present only in financial turmoil. Findings on the positive tail are consistent with herding, while findings on the negative tail are consistent with fire sales.

We also provide some insights into investor behavior that likely lead to market clustering. The persistence of our market clustering measure could be explained by herding and order spreading over time. Market conditions obviously affect trading decisions. We find an indication that the homogeneity of the investors’ pool per stock increases if there is a positive trend in the market or increase in aggregate volatility. However, the volatility effect is short-term and reverses in the month afterwards. Furthermore, we find asymmetries across market conditions. In quiet times, investors prefer less liquid stocks. Consistent with fire sales, less liquid stocks are also traded by more homogeneous groups in downward markets. We discover behavior that is consistent with flight-to-safety within equities in the sense that in high volatility markets large firms attract more crowded attention.

Our analysis contributes to the existing literature on three levels. First, we study the influence of trading behavior on price dynamics using novel granular trading data. To our knowledge, the MiFID data set has not been used for this type of market microstructure research before. Second, the idea and method to measure market clustering and its impact on price instability are new to market microstructure research. The use of complex network theory makes the method suitable for large-scale data. The methodological framework can be extended to study the effects of any feature of the market microstructure. Third, the main contribution is the indication of a causal relation between the market clustering and price instability shown in a dynamic panel data model.

The use of network theory in identifying meaningful motifs in market microstructure research is promising because the model is applicable to all types of market microstructure patterns. First, the influence of trading behavior on price dynamics can be investigated using other microstructure motifs, for example, the influence of the diversification of the investors on the price dynamics of the traded stocks. Differentiation between buy and sell orders would enhance the understanding of the difference in dependence between the positive and negative tail of the price dynamics. The persistence of the market clustering measure—evident in consistent positive dependence on past, lagged values of market clustering—is worthy of further investigation of the time dependence of the configuration of the investors’ pools involved in clustered trades. Moreover, the role of news should be investigated further. It is widely accepted that negative news has a much larger impact compared to positive news. To this end, we should analyze the results on a much shorter time scale to see if common information drives clustering. Second, the method can be used for portfolio holdings data and could, for example, contribute to the literature on price comovements due to common active mutual fund owners [1]. Third, the method can be used to study trading patterns separate from price dynamics, for example, the evolution of clustering patterns over time. Furthermore, the relation between clustering and current market conditions needs further attention, for example, what is the mechanism of spillovers in each case.

## Figures and Tables

**Table 1:** Description of variables.

Variable	Definition
MKTF	Fama and French market factor for Europe, returns in % for a month
VIX	The CBOE Volatility index as a proxy to market conditions, level at the end of a month
MOM	12/6-month average of monthly returns in % at the end of a month
MCAP	Log of market capitalization in $10^{6}$ EUR at the end of a month
ILLIQ	A daily ratio of absolute stock return to its euro volume, averaged over a month, also known as Amihud [47] liquidity measure, to reduce heteroskedasticity we transform as $\log(\mathrm{ratio}+10^{-6})$
PB3	Price-to-book ratio with a 3-month publication lag at the end of a month
DY	12-month trailing dividend yield, in % at the end of a month, we set not available values to 0
LEV3	Ratio of long-term debt to capital with a 3-month publication lag at the end of a month

**Table 2:** Descriptive statistics.

	N.Obs.	Mean	Median	St.dev.	Min	Max	Between	Within
ΔVaR	16216	−0.80	0.00	7.68	−39.10	82.10	0.010	0.356
ΔVaL	16216	−0.50	0.00	7.69	−59.74	98.78	0.011	0.190
CLUST	15896	0.04	0.06	0.28	−1.00	6.58	0.245	0.041
MKTF	16216	1.09	1.02	5.97	−12.33	13.86	0.003	1.000
VIX	16216	21.09	18.38	8.12	11.40	46.35	0.052	1.000
MOM	16204	0.64	0.71	3.48	−18.88	27.71	0.106	0.338
MCAP	16216	7.06	6.99	2.15	0.93	12.21	0.970	0.004
ILLIQ	16051	−3.66	−4.40	4.47	−13.82	12.48	0.957	0.007
PB3	15025	1.95	1.35	2.50	0.05	71.67	0.470	0.010
DY	16216	2.88	2.15	4.53	0.00	157.78	0.303	0.032
LEV3	15916	26.39	25.35	20.42	0.00	159.01	0.813	0.007

For each variable the table presents the number of available observations, mean, median, standard deviation, minimum, maximum, and the proportions of between and within variation. Note that the proportions of variation do not add up to one because the panel is unbalanced.

**Table 3:** Correlations of pooled variables.

	ΔVaR	ΔVaL	CLUST	MKTF	VIX	MOM	MCAP	ILLIQ	PB3	DY	LEV3
ΔVaR		0.000	0.018	−0.203	0.186	−0.204	−0.039	0.047	−0.006	0.063	−0.009
ΔVaL	0.293		0.020	−0.098	0.154	−0.253	−0.053	0.047	−0.010	0.081	−0.005
CLUST	0.020	0.011		0.004	0.102	−0.036	−0.041	0.058	−0.024	0.031	−0.025
MKTF	0.000	0.000	0.613		−0.245	0.026	0.015	−0.018	−0.018	−0.021	0.015
VIX	0.000	0.000	0.000	0.000		−0.315	−0.004	0.033	−0.042	0.127	0.054
MOM	0.000	0.000	0.000	0.001	0.000		0.091	−0.088	0.152	−0.202	−0.023
MCAP	0.000	0.000	0.000	0.050	0.612	0.000		−0.620	0.046	0.096	0.227
ILLIQ	0.000	0.000	0.000	0.027	0.000	0.000	0.000		−0.069	−0.036	−0.165
PB3	0.468	0.208	0.003	0.030	0.000	0.000	0.000	0.000		−0.039	0.015
DY	0.000	0.000	0.000	0.007	0.000	0.000	0.000	0.000	0.000		0.059
LEV3	0.276	0.501	0.002	0.062	0.000	0.003	0.000	0.000	0.065	0.000	

The table presents Pearson correlations of pooled variables in the upper right triangles and the corresponding *p*-values to test for zero coefficient in the lower left triangles.

**Table 4:** Percentage of significant ACFs and PACFs for ΔVaR, ΔVaL, and CLUST.

Lag	ΔVaR		ΔVaL		CLUST	
	ACF	PACF	ACF	PACF	ACF	PACF
1	43.49	43.49	35.69	35.69	38.29	38.29
2	17.10	5.20	18.59	5.58	27.14	11.52
3	5.20	2.97	8.18	2.60	24.16	10.41
4	0.74	0.74	2.23	1.12	18.96	4.83
5	0.37	1.12	1.12	0.74	12.64	2.60
6	0.00	0.37	0.37	1.12	8.92	1.49
7	1.12	0.74	0.00	0.74	5.95	1.12

We obtain the autocorelations and partial autocorrelations for 269 time series per variable in the panel data set. The table contains the percentages of cases with significant coefficients for the first seven lags.

**Table 5:** Testing for a relation between market clustering and price instability—annual window.

		2009	2010	2011	2012	2013	2014
(a)	MAD	==	==	==	==	==	++
	Variance	+=	=+	++	==	++	++
	Skewness	+=	++	++	==	++	++
	Kurtosis	++	++	++	++	++	++
(b)	Hill index neg.	−=	==	=−	==	==	==
	Hill index pos.	−−	−−	−−	==	−−	−−
(c)	Outliers neg.	≠=	==	==	==	==	≠=
	Outliers pos.	≠+	≠+	≠+	==	≠+	≠+

We compare the distributions of the four time series measures (a), the Hill indices of the negative and positive tails (b), and the number of outliers per time series (c) over six years between two groups of stocks: the lowest 33% and the highest 33% of the stocks, ranked according to their market clustering measure. The table shows for each comparison two test results. In panels a and b, the first is the Kolmogorov-Smirnov (KS) test and the second is the Mann-Whitney-Wilcoxon (MWW) test. The critical value is 0.025 for both tests. A “+”/“−”/“=” sign means that the distribution for high market clustering exceeds/undercuts/is equal to the distribution for low market clustering. In panel c the first is the $\chi^{2}$-test (critical value: 0.05) and the second is the MWW test (critical value: 0.025). Contrary to the KS test, the $\chi^{2}$ test results indicates only whether the hypothesis of homogeneity is accepted (“=”) or rejected (“≠”). Note that we do not show 2015 because the comparison with other years would be difficult as we have significantly fewer observations.

**Table 6:** Testing for a relation between market clustering and price instability—2-month window.

	2009						2010					
	1	3	5	7	9	11	1	3	5	7	9	11
MAD	==	+=	==	==	==	=−	−−	=−	++	++	++	=+
Variance	==	++	++	=+	++	==	==	==	++	++	++	++
Skewness	==	==	==	++	==	==	+=	==	==	==	++	++
Kurtosis	++	==	++	++	++	+=	+=	++	==	==	++	++
	**2011**						**2012**					
	**1**	**3**	**5**	**7**	**9**	**11**	**1**	**3**	**5**	**7**	**9**	**11**
MAD	==	==	==	++	==	++	==	==	==	==	==	==
Variance	==	==	==	++	++	++	==	+=	==	==	==	+=
Skewness	++	==	++	++	++	==	==	==	==	==	==	==
Kurtosis	++	++	++	+=	==	==	==	==	==	==	==	==
	**2013**						**2014**					
	**1**	**3**	**5**	**7**	**9**	**11**	**1**	**3**	**5**	**7**	**9**	**11**
MAD	++	==	==	=+	==	++	++	++	++	++	++	==
Variance	++	++	++	++	+=	++	++	++	++	++	++	+=
Skewness	==	==	+=	==	++	==	++	++	==	++	+=	+=
Kurtosis	==	+=	++	==	++	++	++	++	=+	++	++	==

Repetition of Table 5a for time windows of two months instead of one year. Contrary to Table 5, here the critical value is 0.05. The dates in the first line indicate the first month of each time window.

**Table 7:** Relation between market clustering and price instability for stocks mainly traded by investors outside MiFID data set.

	2009	2010	2011	2012	2013	2014
Mean	==	=−	=−	==	−=	==
Variance	==	==	==	==	==	=+
Skewness	==	==	==	==	=−	==
Kurtosis	+=	==	==	==	==	=−

Repetition of Table 5a for the stocks for which less than 10% of the total yearly turnover is covered by the investors in the MiFID data set. This category contains on average 434 stocks, which is 44.5% of the total group of selected stocks.

**Table 8:** Estimation results of dynamic panel data models for the clustering measure.

	Model 1		Model 2		Model 3
GMM IV lags
CLUST	2:75		2:75		2:75
ΔVaR	1:75		1:75		1:75
VIX	1:75		1:75		1:75
${\mathrm{CLUST}}_{t-1}$	0.122 ***		0.125 ***		0.122 ***
	(0.014)		(0.014)		(0.014)
${\mathrm{CLUST}}_{t-2}$	0.075 ***		0.078 ***		0.075 ***
	(0.014)		(0.014)		(0.014)
ΔVaR	0.025		−0.007		0.010
	(0.030)		(0.030)		(0.030)
$\Delta {\mathrm{VaR}}_{t-1}$	0.080 **		0.046		0.064 **
	(0.033)		(0.032)		(0.032)
MKTF	0.097 **		0.044		0.241***
	(0.048)		(0.049)		(0.064)
MKTF${}_{t-1}$	0.172 ***		0.116 ***		0.175 ***
	(0.042)		(0.045)		(0.041)
VIX	0.082		−0.028		0.165 ***
	(0.064)		(0.080)		(0.064)
VIX${}_{t-1}$	0.305 ***		0.333 ***		0.302 ***
	(0.093)		(0.094)		(0.092)
VIX${}_{t-2}$	−0.282 ***		−0.245 ***		−0.276 ***
	(0.063)		(0.066)		(0.063)
MOM	−0.105	MOMhigh	−0.234	MOMup	−0.097
	(0.121)		(0.185)		(0.118)
MCAP	0.339 **	MOMlow	0.017	MOMdown	−0.055
	(0.168)		(0.122)		(0.146)
ILLIQ	0.277 **	MCAPhigh	0.734 ***	MCAPup	−0.127
	(0.108)		(0.249)		(0.156)
		MCAPlow	0.367 **	MCAPdown	0.235
			(0.175)		(0.148)
		ILLIQhigh	0.133	ILLIQup	0.159
			(0.134)		(0.113)
		ILLIQlow	0.346 ***	ILLIQdown	0.318 ***
			(0.115)		(0.104)
No. IVs	237		243		243
Sargan stat	246.290		245.255		245.197
DF	225		228		228
*p*-value	0.157		0.206		0.207
AR(1)	0.000		0.000		0.000
AR(2)	0.383		0.321		0.350
corr${}^{2}\left(y,\hat{y}\right)$	0.198		0.191		0.197

This table contains estimation results of Equation (6) using a two-step system GMM approach with collapsed GMM-style instruments. The dependent variable is the clustering measure. Coefficients for the price-to-book ratio (PB3), trailing dividend yield (DY), and the leverage ratio (LEV3) are insignificant in all models and are not reported to conserve space. Here, CLUST is multiplied by 100, and the number of stock-month observations is 27031. Variables with suffixes “high”, “low”, “up”, and “down” are interacted with 𝟙_(VIX≥25)_, 𝟙_(VIX<25)_, 𝟙_(MKTF≥0)_, and 𝟙_(MKTF<0)_, respectively. Standard errors are in parentheses below the estimates. Coefficients significant at 5, and 1% level are marked with **, and ***, respectively. Obvious subscripts *s* and *t* are omitted for brevity. At the end of the table, usual dynamic panel data model diagnostics are provided: Sargan’s test and *p*-values for Arellano–Bond test for serial correlation. ${\mathrm{corr}}_{y,\hat{y}}^{2}$ measures squared correlation between the dependent variable and the fitted values from the model.

**Table 9:** Estimation results of dynamic panel models for ΔVaR and ΔVaL.

	ΔVaR		ΔVaL	
	Model 1	Model 2	Model 3	Model 4
$y_{t-1}$	0.136 ***	0.131 ***	0.103 ***	0.100 ***
	(0.012)	(0.013)	(0.017)	(0.017)
$y_{t-2}$	0.095 ***	0.090 ***	0.073 ***	0.071 ***
	(0.011)	(0.011)	(0.011)	(0.011)
CLUST	1.598		6.150 ***	
	(1.209)		(1.462)	
CLUST${}_{t-1}$	0.179		−0.192	
	(0.401)		(0.387)	
CLUSTlow		1.398		3.055 **
		(1.352)		(1.44)
CLUSTlow${}_{t-1}$		−0.079		−0.001
		(0.469)		(0.481)
CLUSThigh		5.315 **		7.341 ***
		(2.176)		(2.121)
CLUSThigh${}_{t-1}$		1.239		−0.214
		(0.975)		(0.701)
MKTF	−0.303 ***	−0.307 ***	−0.047 ***	−0.050 ***
	(0.016)	(0.016)	(0.016)	(0.015)
MKTF${}_{t-1}$	−0.144 ***	−0.146 ***	−0.107 ***	−0.105 ***
	(0.010)	(0.010)	(0.011)	(0.011)
VIX	0.117 ***	0.095 ***	0.158 ***	0.141 ***
	(0.024)	(0.027)	(0.024)	(0.026)
VIX${}_{t-1}$	0.009	0.01	−0.113 ***	−0.107 ***
	(0.023)	(0.024)	(0.023)	(0.024)
MOM	−0.303 ***	−0.301 ***	−0.416 ***	−0.412 ***
	(0.030)	(0.030)	(0.036)	(0.034)
MCAP	−0.337 ***	−0.294 ***	−0.213 ***	−0.175 ***
	(0.038)	(0.043)	(0.046)	(0.049)
ILLIQ	−0.060 ***	−0.049 ***	−0.046 ***	−0.027
	(0.015)	(0.016)	(0.018)	(0.017)
PB3	0.008	0.013	0.087	0.085
	(0.053)	(0.051)	(0.056)	(0.053)
DY	0.016	0.019	0.013	0.022
	(0.023)	(0.022)	(0.033)	(0.029)
LEV3	−0.008 **	−0.007 **	−0.001	0.000
	(0.003)	(0.003)	(0.004)	(0.003)
No. IVs	241	316	241	316
Sargan stat	239.556	242.341	240.421	237.452
DF	227	300	227	300
p-value	0.271	0.994	0.258	0.997
AR(1)	0.000	0.000	0.000	0.000
AR(2)	0.265	0.146	0.355	0.504
corr${}_{y,\hat{y}}^{2}$	0.151	0.150	0.070	0.083

This table contains the estimation results of Equation (6) using a two-step system GMM approach. All available lags for dependent variable, (conditional) CLUST, and VIX are used as collapsed GMM instruments. Here, the number of stock-month observations is 27295. Models 1 and 2 use dependent variable $y_{st}=\Delta {\mathrm{VaR}}_{st}$ and Models 3 and 4 use $y_{st}=\Delta {\mathrm{VaL}}_{st}$. Models 2 and 4 introduce $\mathrm{CLUSTlow}=\mathrm{CLUST}\times {𝟙}_{\left(\mathrm{VIX}<25\right)}$ and $\mathrm{CLUSThigh}=\mathrm{CLUST}\times {𝟙}_{\left(\mathrm{VIX}\geq 25\right)}$ to account for asymmetric effects. Standard errors are in parentheses below the estimates. Coefficients significant at 5, and 1% level are marked with **, and ***, respectively. Obvious subscripts s and t are omitted for brevity. At the end of the table, usual dynamic panel data model diagnostics are provided: Sargan’s test and *p*-values for Arellano–Bond test for serial correlation. ${\mathrm{corr}}_{y,\hat{y}}^{2}$ measures squared correlation between the dependent variable and the fitted values from the model.

## Data Availability

The study uses data from MIFID as explained in Section 4. This data is confidential and cannot be published.

## Appendix A. Construction of the Maximum-Entropy Ensemble of Networks with Given Expected Degree Sequence

We define a bipartite network that describes the aggregate trading behavior during a particular month. For each month, *t*, the investment behavior comprised by the data is represented in a binary bipartite graph. The bipartite network has two (say, bottom and top) layers with edges between the two layers. No edges occur between two nodes in the same layer. The nodes of the first (say, bottom) layer represent the set $S_{t}$ of securities that are traded during month *t*. The nodes of the second (say, top) layer represent the set $F_{t}$ of firms that perform trades during month *t*. The individual securities are indicated by the label *s* each month separately, such that $s\in S_{t}$. The label can take the values $s=1,...,n_{S,t}$ with $n_{S,t}=\left|S_{t}\right|$ the number of elements in $S_{t}$. Similarly, the individual firms are indicated by the label *f* each month separately, such that $f\in F_{t}$. The label can take the values $f=1,...,n_{F,t}$ with $n_{F,t}=\left|F_{t}\right|$ the number of elements in $F_{t}$.

The performed transactions are represented by the edges between the firms and the securities, denoted by the rectangular binary adjacency matrix (sometimes called “bi-adjacency matrix”) $A_{t}$ with elements $a_{sf,t}$. The size of matrix $A_{t}$ is $n_{S,t}\times n_{F,t}$. The transactions are represented as follows: $a_{sf,t}=1$ in case firm *f* traded in security *s* during month *t* and $a_{sf,t}=0$ otherwise. The observed degree of firm *f* during month *t* is given by

(A1) $$d_{f,t}^{\mathrm{obs}}=\sum_{s=1}^{n_{S,t}}a_{sf,t}$$

and the observed degree of security *s* is given by

(A2) $$d_{s,t}^{\mathrm{obs}}=\sum_{f=1}^{n_{F,t}}a_{sf,t}.$$

The set $D_{t}^{\mathrm{obs}}$ contains the observed degrees of all nodes in month *t*, such that

(A3) $$d_{s,t}^{\mathrm{obs}},d_{f,t}^{\mathrm{obs}}\in D_{t}^{\mathrm{obs}}\;\forall f,s$$

Note that the graph indicates only whether a trade of a firm in a security occurs. None of the following quantities are represented: the number of transactions, the number of underlying securities, or the turnover. Furthermore, the graph does not distinguish between buy and sell transactions or between agency and principal transactions.

For a given *t*, our goal is to find the probability distribution $P_{t}\left(X_{t}\right)=P\left(X_{t}|D_{t}^{\mathrm{obs}}\right)$ over an allowed set of alternative trading configurations, such that the ensemble of bipartite graphs generated by $P_{t}$ is maximally random, apart from ensuring that the expected value $\langle D_{t}\rangle$ of the degree sequence under $P_{t}$ equals the observed value $D_{t}^{\mathrm{obs}}$, i.e.,

(A4) $$\langle D_{t}\rangle =D_{t}^{\mathrm{obs}}.$$

This prescription ensures that, besides the information about the observed degree sequence, all other empirical information about the actual placing of the trades is not used to determine $P_{t}$ and cannot be retrieved form it. To further ensure that the inference of higher-order properties obtained using $P_{t}$ is unbiased, we apply the maximum-entropy method [13] and look for the distribution $P_{t}\left(X_{t}\right)$ that maximizes Shannon’s entropy functional

(A5) $$S_{t}\left[P_{t}\right]=-\sum_{X_{t}\in G_{t}}P_{t}\left(X_{t}\right)\ln P_{t}\left(X_{t}\right)$$

where the sum runs over the ensemble of graphs $G_{t}$ containing all binary, bipartite networks where the number of elements in the top layer is $n_{F,t}$ and the number elements in the bottom layer is $n_{S,t}$. The resulting ensemble is a canonical one [13], which means that all the allowed graphs have the same number of nodes as the original empirical network but the number of links varies between zero and $n_{S,t}\;n_{F,t}$. An element $X_{t}\in G_{t}$ is a $n_{S,t}\times n_{F,t}$ adjacency matrix encoding the configuration of a possible bipartite network in the ensemble. There are $2^{n_{S,t}n_{F,t}}$ possible such configurations. Each configuration $X_{t}$ contains, for each security–firm pair (s,f), the information whether the firm trades the security ($a_{sf}\left(X_{t}\right)=1$) or not ($a_{sf}\left(X_{t}\right)=0$) during month *t*. $X_{t}$ does not denote the observed graph configuration, but a generic allowed configuration in $G_{t}$. Among these configurations, a particular one $X_{t}^{\mathrm{obs}}$ is the observed one, i.e., $a_{sf}\left(X_{t}^{\mathrm{obs}}\right)=a_{sf,t}$ for all s,f.

Shannon’s entropy can be seen as the “degree of uncertainty” encoded in the probability distribution $P_{t}$ and is a weighted average of the amount of information required to identify a specific graph in the ensemble. For example, in case of no constraints, Shannon’s entropy would be maximized when each configuration $X_{t}$ occurs with equal probability $P_{t}\left(X_{t}\right)=2^{-n_{S,t}n_{F,t}}$ and its value would be $S_{t}\left[P_{t}\right]=\ln2^{n_{S,t}n_{F,t}}$. In our case, or a given month *t*, we instead need to maximize $S_{t}\left[P_{t}\right]$ under the constraints imposed by the degree sequence, i.e., Equation (A4), which we rewrite as

(A6) $$\langle d_{f,t}\rangle =d_{f,t}^{\mathrm{obs}},\;\;\langle d_{s,t}\rangle =d_{s,t}^{\mathrm{obs}}\;\forall f,s,$$

where

(A7) $$\langle d_{f,t}\rangle =\langle d_{f}\left(X_{t}\right)\rangle =\langle \sum_{s=1}^{n_{S,t}}a_{sf}\left(X_{t}\right)\rangle ,\;\langle d_{s,t}\rangle =\langle d_{s}\left(X_{t}\right)\rangle =\langle \sum_{f=1}^{n_{F,t}}a_{sf}\left(X_{t}\right)\rangle .$$

Note that in total there are $n_{F,t}+n_{S,t}+1$ constraints for each *t*:

(A8) $$\left\{\begin{array}{c} d_{f,t}^{\mathrm{obs}}=\sum_{X_{t}\in G_{t}}P_{t}\left(X_{t}\right)d_{f}\left(X_{t}\right)\;\;\;\forall f,\\ d_{s,t}^{\mathrm{obs}}=\sum_{X_{t}\in G_{t}}P_{t}\left(X_{t}\right)d_{s}\left(X_{t}\right)\;\;\;\forall s,\\ 1=\sum_{X_{t}\in G_{t}}P_{t}\left(X_{t}\right), \end{array}\right.$$

where the last expression is the normalization of the probability distribution. We therefore introduce $n_{F,t}+n_{S,t}+1$ Lagrange multipliers ${\left\{\beta_{f,t}\right\}}_{f=1}^{n_{F,t}}$, ${\left\{\beta_{s,t}\right\}}_{s=1}^{n_{S,t}}$, $\alpha_{t}$ (one for each constraint) and look for the probability distribution $P_{t}$ optimizing the Lagrange function

(A9) $$\begin{array}{ccc} L_{t}\left[P_{t}\right] & = & S_{t}\left[P_{t}\right]+\alpha_{t}\left(1-\sum_{X_{t}\in G_{t}}P_{t}\left(X_{t}\right)\right)\\ & & +\sum_{f=1}^{n_{F,t}}\beta_{f,t}\left(d_{f,t}^{\mathrm{obs}}-\sum_{X_{t}\in G_{t}}P_{t}\left(X_{t}\right)d_{f}\left(X_{t}\right)\right)\\ & & +\sum_{s=1}^{n_{S,t}}\beta_{s,t}\left(d_{s,t}^{\mathrm{obs}}-\sum_{X_{t}\in G_{t}}P_{t}\left(X_{t}\right)d_{s}\left(X_{t}\right)\right). \end{array}$$

Taking the functional derivative, we get

(A10) $$\frac{\delta L_{t}}{\delta P_{t}\left(X_{t}\right)}=\ln P_{t}\left(X_{t}\right)+1+\alpha_{t}+\sum_{f=1}^{n_{F,t}}\beta_{f,t}d_{f}\left(X_{t}\right)+\sum_{s=1}^{n_{S,t}}\beta_{s,t}d_{s}\left(X_{t}\right).$$

Now the probability distribution $P_{t}\left(X_{t}\right)=P\left(X_{t}|D_{t}^{\mathrm{obs}}\right)$ is determined by the optimum:

(A11) $$\frac{\delta L_{t}}{\delta P_{t}\left(X_{t}\right)}=0\;\;↔\;\;P\left(X_{t}|D_{t}^{\mathrm{obs}}\right)=\frac{e^{-H_{t}\left(X_{t}\right)}}{Z_{t}},$$

with $H_{t}\left(X_{t}\right)$ the so-called Hamiltonian

(A12) $$H_{t}\left(X_{t}\right)=\sum_{f=1}^{n_{F,t}}\beta_{f,t}d_{f}\left(X_{t}\right)+\sum_{s=1}^{n_{S,t}}\beta_{s,t}d_{s}\left(X_{t}\right),$$

and $Z_{t}$ the so-called partition function

(A13) $$Z_{t}=e^{1+\alpha_{t}}=\sum_{X_{t}\in G_{t}}e^{-H_{t}\left(X_{t}\right)}.$$

The partition function can be written as [13]

(A14) $$\begin{array}{cc} Z_{t} & =\sum_{X_{t}\in G_{t}}e^{-\sum_{f}\beta_{f,t}\sum_{s}a_{sf}\left(X_{t}\right)-\sum_{s}\beta_{s,t}\sum_{f}a_{sf}\left(X_{t}\right)} \end{array}$$

(A15) $$\begin{array}{cc} & =\sum_{X_{t}\in G_{t}}\prod_{s,f}e^{-\beta_{f,t}a_{sf}\left(X_{t}\right)-\beta_{s,t}a_{sf}\left(X_{t}\right)} \end{array}$$

(A16) $$\begin{array}{cc} & =\prod_{s,f}\left(1+e^{-\beta_{f,t}-\beta_{s,t}}\right). \end{array}$$

Now, we rewrite $P(X_{t}|D_{t}^{\mathrm{obs}})$ in a factorized form that shows the probabilistic independence of all edges of the network (note that this independence is not an assumption or simplification, as it follows mathematically from our choice of the constraints):

(A17) $$\begin{array}{cc} P(X_{t}|D_{t}^{\mathrm{obs}})\; & =\prod_{s,f}p_{sf,t}^{a_{sf}\left(X_{t}\right)}{\left(1-p_{sf,t}\right)}^{1-a_{sf}\left(X_{t}\right)}, \end{array}$$

where we have introduced the security-firm connection probability

(A18) $$p_{sf,t}=P\left(a_{sf}\left(X_{t}\right)=1|D_{t}^{\mathrm{obs}}\right)=\frac{x_{f,t}x_{s,t}}{1+x_{f,t}x_{s,t}},$$

the complementary (no connection) probability

(A19) $$1-p_{sf,t}=P\left(a_{sf}\left(X_{t}\right)=0|D_{t}^{\mathrm{obs}}\right)=\frac{1}{1+x_{f,t}x_{s,t}},$$

and the reparametrization

(A20) $$\left\{\begin{array}{c} x_{f,t}=e^{-\beta_{f,t}}\\ x_{s,t}=e^{-\beta_{s,t}} \end{array}\right..$$

The variables $x_{f,t}$ and $x_{s,t}$ are also called “hidden variables” [13]. Their numerical value is found by solving, for each *t*, the $n_{F,t}+n_{S,t}$ coupled nonlinear Equation (A6) realizing the value of the imposed constraints. Noticing that

(A21) $$\langle a_{sf}\left(X_{t}\right)\rangle =\sum_{X_{t}\in G_{t}}P_{t}\left(X_{t}\right)a_{sf}\left(X_{t}\right)=p_{sf,t},$$

those equations can be rewritten explicitly in terms of the hidden variables as follows:

(A22) $$\sum_{s=1}^{n_{S,t}}\frac{x_{f,t}x_{s,t}}{1+x_{f,t}x_{s,t}}=d_{f,t}^{\mathrm{obs}},\;\sum_{f=1}^{n_{F,t}}\frac{x_{f,t}x_{s,t}}{1+x_{f,t}x_{s,t}}=d_{s,t}^{\mathrm{obs}}\;\forall f,s.$$

It can be proven in general [13] that the values solving the above equations are unique and correspond to the values that maximize the likelihood $P_{t}\left(A_{t}\right)=P\left(A_{t}|D_{t}^{\mathrm{obs}}\right)$ of generating the observed network $A_{t}$, given the model parameters. Various efficient codes are available for solving the above type of equations [48].

## Appendix B. Stochastic Dominance of Price Instability Measures

In this appendix, we report (see Figure A1 and Figure A2) the cumulative distributions, for low and high market clustering, for all the time series measures (MAD, variance, skewness, kurtosis, number of negative outliers, number of positive outliers, Hill index for the negative tail, and Hill index for the positive tail) and all years.

## Appendix C. Robustness Checks

Table A1 shows the results for the relation between market clustering and the kurtosis for various segments of the log return distribution. The results for the kurtosis do not depend in particular on the tails of the log return distribution.

**Table A1:** The kurtosis results from Table 5 for partial data.

	2009	2010	2011	2012	2013	2014
10–90	++	++	++	++	++	+=
20–80	++	++	++	++	++	==
30–70	++	++	++	++	++	==
40–60	++	=+	++	=+	++	++

First, we order the log returns time series per stock and per year in ascending order. Second, we select the segments of the log return distribution as shown in the left column (in percentages). For example, the last line shows the results for the segment 40–60% (the middle part), which means that we remove the first 40% and the last 40% of the ordered log return distribution.

Table A2 shows the relation between market clustering and the kurtosis for different cross-sections of the market clustering distribution. The critical value is 0.025 in all tables. The results show no consistent variation over the different cross sections.

**Table A2:** The relation between market clustering and the kurtosis for different cross sections of the market clustering distribution.

	2009	2010	2011	2012	2013	2014
0–10 and 90–100	++	==	+=	++	++	++
10–30 and 70–90	++	==	++	==	=+	++
0–50 and 50–100	++	++	+=	++	++	++
20–50 and 50–80	==	=+	==	==	++	=+

For comparison, Table 5 shows the results for the highest 33% and the lowest 33% of the stocks, ranked according to their market clustering measure, i.e., the selected regions are 0–33% and 67–100%.

Table A3 shows the result for normalization by the time-varying standard deviation, estimated by various GARCH-type models. Normalization by the time-varying volatility means that the weight of the price fluctuations in periods of high volatility is effectively reduced in favor of the weight of the price fluctuations during tranquil periods. We estimate for each stock the conditional volatility time series for the complete log return time series at once instead of each year separately. The EGARCH model allows the sign and the magnitude of the log returns to have separate effects on the volatility. In the GJR-GARCH model, the effects of the positive and negative log returns are estimated separately. The EGARCH models are exponential and therefore less sensitive to outliers than the GJR-GARCH models. The addition of extra lags allows the volatility to vary on both shorter and longer time scales. For all GARCH models we we assume conditional normal distribution for the error term: $\varepsilon_{s,t}∼N\left(0,\sigma_{s,t}^{2}\right)$. This assumption is probably violated for some of the stocks. We assume that the consequences of this violation are limited.

The relation between market clustering and price instability remains consistently positive when we account for the time-varying volatility. We find no apparent variation of the results for the relation between market clustering and price instability for the different GARCH models for the conditional volatility. Apparently, market clustering causes downward price shocks not only during volatile periods but also when the price is more stable.

**Table A3:** The results for the yearly kurtosis of the log returns, normalized by the conditional standard deviation estimated by various GARCH models.

	2009	2010	2011	2012	2013	2014
GARCH(1,1)	++	++	==	++	++	++
GARCH(2,2)	++	==	==	++	++	++
EGARCH(1,1)	++	++	==	++	++	++
GJR-GARCH(1,1)	++	++	==	++	++	++
